# Supplementary material for: Self-reported body weight and weight-related stigmatization experiences among young adult women—two contexts, but similar attitudes related to body image, mental self-schemas, self-esteem, and stereotypes of people with obesity
Source: PeerJ. 2021 Sep 27;9:e12047. doi: 10.7717/peerj.12047 (PMC8483002; doi:10.7717/peerj.12047)
Supplement: Supplemental Information 1 [file peerj-09-12047-s001.docx]

**Table S1. Body image, self-esteem and mental self-schemas in groups with BMI indicating normal weight, overweight, and obesity – Kruskal-Wallis H tests**

| Dependent variable | H (df = 2; n = 374); p-value | Mean rank | | |
| --- | --- | --- | --- | --- |
|  |  | Normal weight  (n = 280) | Overweight  (n = 53) | Obesity  (n = 27) |
| (1) *Actual* body image | **137.756; p < .001*** | 154.62ab | 287.30a(c) | 349.59b(c) |
| (2) *Ideal* body image | **36.192; p < .001*** | 171.53ab | 230.02a | 277.91b |
| (3) *Reflected* body image | **135.989; p < .001*** | 154.74ab | 286.94a(c) | 348.96b(c) |
| (4) *Ought* body image according to men | .287; p = .867 | 186.74 | 194.12 | 184.78 |
| (5) *Ought* body image according to women | 1.090; p = .580 | 187.95 | 194.36 | 169.09 |
| Discrepancy (1)–(2) | **82.903; p < .001*** | 162.12ab | 266.25a | 309.30b |
| Discrepancy (1)–(3) | 9.169; p = .010 | 195.58 | 161.37 | 150.80 |
| Discrepancy (1)–(4) | **111.091; p < .001*** | 157.96ab | 277.08a | 333.33b |
| Discrepancy (1)–(5) | **112.180; p < .001*** | 158.29ab | 270.62a(c) | 342.37b(c) |
| (6) Mental *actual self* | 9.955; p = .007 | 196.61(a) | 157.91(b) | 146.59 |
| (7) Mental *reflected self* | 8.164; p = .017 | 194.88(a) | 171.85 | 137.91(a) |
| Discrepancy (6)–(7) | 1.350; p = .509 | 187.04 | 179.28 | 208.69 |
| Self-esteem | 6.903; p = .032 | 194.84 | 166.96 | 147.94 |
| The stereotype of a person with obesity | **23.956; p < .001*** | 200.71(a)b | 154.79(a) | 107.83b |

* – p-value significant with the Holm-Bonferroni correction.

The same letters next to mean ranks indicate significant differences in the post-hoc test. Letters in parentheses indicate p-value < .050. Letters without parentheses indicate p-value < .001.
