# Supplementary material for: Self-reported body weight and weight-related stigmatization experiences among young adult women—two contexts, but similar attitudes related to body image, mental self-schemas, self-esteem, and stereotypes of people with obesity
Source: PeerJ. 2021 Sep 27;9:e12047. doi: 10.7717/peerj.12047 (PMC8483002; doi:10.7717/peerj.12047)
Supplement: Supplemental Information 2 — Explains the titles of variables in the raw data. [file peerj-09-12047-s002.docx]

| ID | participant |
| --- | --- |
| Age | age |
| Height | height (in metres) |
| Mass | body mass (in kilograms) |
| BMI | body mass index (Mass/Height^2) |
| WEIGHT_NORM | weight status; “YES” if BMI value was between 18.5 and 24.9; “NO” if BMI<24.9 |
| FAT_1 | “Do you think you are currently fat?” |
| FAT_2 | “Are you currently ashamed of how much you weigh?” |
| STIGMA | weight-related stigmatization experiences; “NO” if values of FAT_1 and FAT_2 are “NO”; “YES” if at least one of values of FAT_1 and FAT_2 is “YES” |
| (1)_ABI | actual body image; the value related to picture from The Contour Drawing Rating Scale |
| (2)_IBI | Ideal body image; the value related to picture from The Contour Drawing Rating Scale |
| (3)_RBI | reflected body image; the value related to picture from The Contour Drawing Rating Scale |
| (4)_OBIAM | ought body image according to men; the value related to picture from The Contour Drawing Rating Scale |
| (5)_OBIAW | ought body image according to women; the value related to picture from The Contour Drawing Rating Scale |
| DIS_(1)-(2) | discrepancy between physical actual body image and ideal body image |
| DIS_(1)-(3) | discrepancy between physical actual body image and reflected body image |
| DIS_(1)-(4) | discrepancy between physical actual body image and ought body image according to men |
| DIS_(1)-(5) | discrepancy between physical actual body image and ought body image according to women |
| SkRAP_actual_1  …  SkRAP_actual_12 | values of responses from Self-Discrepancy Questionnaire (SkRAP) related to psychic actual self |
| SkRAP_reflected_1  …  SkRAP_reflected_12 | values of responses from Self-Discrepancy Questionnaire (SkRAP) related to psychic reflected self |
| (6)_MAS | mental actual self; the sum of values from SkRAP_actual_1 to SkRAP_actual_12 |
| (7)_MRS | mental reflected self; the sum of values from SkRAP_reflected_1 to SkRAP_reflected_12 |
| DIS_(6)-(7) | discrepancy between mental actual self and psychic ideal self |
| SES_1  …  SES_10 | responses to items from Rosenberg’s Self Esteem Scale (SES); values of responses to items marked with “*” have to be inverted before counting the total score (“1” to “4”; “2” to “3”; “3” to “2” and “4” to “1”) |
| SES_GS | general score of Rosenberg’s Self Esteem Scale (SES); the sum of item scores from SES_1 to SES_10 after necessary inversions of scores marked with “*” |
| OPS_1_...  …  OPS_10_... | values of responses to items related to obese person stereotype |
| OPS_GS | general score related to obese person stereotype; the mean value of item scores from OPS_1_... to OPS_10_... |
